# Significance of PRO2000/ANCCA expression, a novel proliferation-associated protein in hepatocellular carcinoma

**DOI:** 10.1186/1475-2867-14-33

**Published:** 2014-04-04

**Authors:** Jie Yang, Jie Huang, Luqiao Luo, Zhenzhu Chen, Ying Guo, Linlang Guo

**Affiliations:** 1 Department of Pathology, Zhujiang Hospital, Southern Medical University, Guangzhou, China; 2 Department of Organ Transplantation, Zhujiang Hospital, Southern Medical University, Guangzhou, China

**Keywords:** PRO2000/ANCCA, Proliferation, Hepatocellular carcinoma (HCC), Immunohistochemistry

## Abstract

**Background:**

PRO2000/ANCCA may be an important candidate gene which located within a region of chromosome 8q in hepatocellular carcinoma (HCC). However, its significance remains unclear. The aim of this study was to explore the clinical significance of PRO2000/ANCCA expression in HCC.

**Methods:**

The correlations of PRO2000/ANCCA expression with clinicopathological factors and prognosis of HCC patients were analyzed. Expression of PRO2000/ANCCA, ki-67, cyclinD1, p53 and p21 was detected in HCCs from 107 patients along with corresponding non-tumor tissues by immunohistochemistry.

**Results:**

PRO2000/ANCCA expression was present in 66 of 107 (64.94%) HCC specimens in which 36 of 76 (47.37%) in well differentiated tumors and 30 of 31 (96.77%) in poorly differentiated tumors respectively, while 8 (7.48%) in adjacent non-tumor tissues with scattered positive cells. PRO2000/ANCCA expression was associated with clinicopathological features such as histological differentiation, number of tumor nodules, TNM stage, tumor microsatellite, portal vein tumor thrombus and recurrence, but not with gender, age, tumor size, cirrhosis, HBV infection and serum fetoprotein (AFP) level. There was a close relationship between PRO2000/ANCCA and ki-67 and cyclinD1 in HCC. PRO2000/ANCCA immunopositivity was independent of p53 and p21^WAF1/Cip1^.

**Conclusions:**

Increased expression of PRO2000/ANCCA is associated with adverse outcome in patients with HCC and is a predictor of poor prognosis for HCC. PRO2000/ANCCA may be involved in the development of HCC and might promote cell proliferation through a p53/ P21^WAF1/Cip1^-independent pathway.

## Introduction

Primary hepatocellular carcinoma (HCC), is the fifth most common cancer worldwide and the third most common cause of cancer-related mortality with approximately 696,000 deaths each year. More than 50% of the worldwide cases of HCC occur in China, because of the high prevalence of chronic hepatitis B virus infection and liver cirrhosis [1,2]. A detailed understanding of the molecular mechanisms associated with HCC ultimately could improve our current diagnosis and treatments for this disease. Throughout the years, many important advances have been made to understand the pathogenesis of HCC. A subset of cytogenetic changes, including frequent gain of chromosome 8q which the most commonly amplified region in multiple cancer types were found to be involved in the early development of HCC by comparative genomic hybridization (CGH) and comparative genomic microarray analysis (CGMA). PRO2000/ANCCA, one of the genes on chromosome 8q was highly expressed (4.7 folds) in HCC comparing to corresponding non-cancerous samples and showed a high correlation between DNA copy number and expression levels in HCC samples. The data suggested that PRO2000/ANCCA may be an important candidate gene in the development of HCC [3].

PRO2000, a novel member of the AAA + superfamily, has been also named as ANCCA (AAA nuclear coregulator cancer-associated protein) or ATAD2 (AAA domain containing 2) or TAAB (ACTR target with AAA + ATPase and bromodomain) in the NCBI protein database [4]. ANCCA and ATAD2 are the most commonly used names in the published data, but we prefer to call it PRO2000, which is the originate name of this gene first discovered in HCC. PRO2000/ANCCA contains two AAA + domains in the central region with the first one being crucial for its transcription coactivator function and a bromodomain close to the COOH-terminus that specifically recognizes acetylated histones [5]. Several laboratories have reported that PRO2000/ANCCA was overexpressed in different human cancers, including breast cancer, prostate cancer, lung cancer and endometrial cancer. High levels of PRO2000/ANCCA was associated with poor prognosis in cancer patients. These studies showed PRO2000/ANCCA was involved in the estrogen and androgen receptor pathways to mediate estrogen- or androgen-induced expression of specific subsets of proliferation-associated genes. In addition, PRO2000/ANCCA acts as an important co-regulator of MYC and contributes to the development of aggressive cancer [6-13]. However, the molecular mechanisms of PRO2000/ANCCA leading to hepatocarcinogenesis and progression are still not clearly understood.

In this study, we investigated for the first time the significance of PRO2000/ANCCA expression in HCC. Protein expression of PRO2000/ANCCA was examined in relation to clinicopathological features in 107 cases of HCC by immunohistochemical technique. The relationships between PRO2000/ANCCA expression and ki-67, cyclin D1, p53 and p21^WAF1/Cip1^ were also analyzed in the present study.

## Materials and methods

### Tissue preparation

One hundred and seven samples were obtained by surgical resection in our department between January 2004 and June 2008. The patients, 96 males (89.7%) and 11 females (10.3%), ranged in age from 30 to 79 years (mean 51.45). Tumor sizes were divided as less than or equal to 5 cm (n = 55) and more than 5 cm (n = 52). Tumor stage was defined according to the 7th edition of the American Joint Committee on Cancer staging manual. Written informed consent was obtained from all the patients for the use of the tumor tissues for clinical research and the publication of this report. The project protocol was approved by the Institutional Ethics Committee of Zhujiang Hospital prior to the initiation of the study. Clinicopathological features of study population were presented in Table 1. All samples were independently reviewed by two pathologists. The cases of HCC were classified according to the criteria described by Edmondso-Steiner and grouped as well differentiated (gradeI-II; n = 76) or poorly differentiated (grade III-IV; n = 31). All 107 specimens contained pericarcinomatous tissues, in which including 56 cases with cirrhosis. Sixty-three patients were seropositive for HBsAg and HBeAg. No positive case for HCV was present in this study. All tissues were fixed in 10% formalin (pH 7.0) for 12–24 hours and embedded in paraffin wax and then 4 μm serial sections were cut and mounted on poly-l-lysine coated slides.

**Table 1:** Clinicopathological correlation of PRO2000/ANCCA expression in HCC

**Feature**	**Cases**	**PRO2000/ANCCA**			***P*-value**
		**-**	**+**	**++**	
**Gender**					0.697
Male	96	38	46	12	
Female	11	3	6	2	
**Age (years)**					0.524
≤51	53	23	23	7	
>51	54	18	29	7	
**Serum HBsAg**					0.737
Negative	44	17	20	7	
Positive	63	24	32	7	
**Serum AFP (ng/ml)**					0.703
≤20	48	18	25	5	
>20	59	23	27	9	
**Tumor size (cm)**					0.107
≤5	55	25	26	4	
>5	52	16	26	10	
**Tumor number**					**0.003**
Single	80	38	32	10	
Multiple	27	3	20	4	
**Tumor differentiation**					**<0.001**
I-II	76	40	34	2	
III-IV	31	1	18	12	
**Cirrhosis**					0.519
Negative	51	21	22	8	
Positive	56	20	30	6	
**TNM stage**					**<0.001**
I-II	81	41	38	2	
III-IV	26	0	14	12	
**Tumor microsatellite**					**0.011**
Absent	77	36	31	10	
Present	30	5	21	4	
**Portal vein tumor thrombus**					**<0.001**
Absent	96	41	47	8	
Present	11	0	5	6	
**Recurrence**					**<0.001**
Absent	89	39	43	7	
Present	18	2	9	7	

-, negative; +, moderate positive; ++, strong positive. Significant differences are shown in bold.

### Immunohistochemical staining

Sections were deparaffinized and rehydrated routinely. Before adding the primary antibody, antigen was retrieved by heating sections in 10 mM citrate buffer (pH 6.0) in a microwave oven for 10 minutes followed by 10 minutes of cooling. After blocking with 0.3% H_2_O_2_ and goat serum, the slides were then incubated with a primary antibody, directed against PRO2000/ANCCA (1:100 dilution, gift from Dr Hongwu Chen’s lab, UCDAVIS, Cancer center, USA), ki-67(MIB-1, Dako, Glostrup, Denmark), cyclin D1(SP4, Dako, Glostrup, Denmark), p53(DO-7, Dako, Glostrup, Denmark) and p21^WAF1/Cip1^(4D10, Dako, Glostrup, Denmark) at 4°C overnight. Biotinylated secondary antibodies were then applied according to the manufacturer’s recommendations (Amersham). After incubation with avidin biotin complex using the Vector Elite ABC detection kit (Vector Labs, Burlingame, USA), reaction products were visualized by 3’diaminobenzidine (DAB), and slides were subsequently counterstained with hematoxylin. Brown-yellow granules in nucleus or cytoplasm were considered positive staining. The positive reactivity was scored semiquantitatively by microscopic evaluation according to the estimated number of positive nuclei or cytoplasm of target cells. The scores were graded into four groups from 0 to 3+ as follow : 0, no positive cells; +, less than 25% of positive cells; 2+, 25-50% of positive cells; and 3+, >50% of positive cells. Negative controls were performed by replacing the primary antibodies stated above with PBS. The specimens were classified according to IHC staining scores of PRO2000/ANCCA as negative group (−, n = 41), moderate positive group (+, n = 52), strong positive group (++, n = 14).

### Statistical analysis

Correlation of PRO2000/ANCCA expression with clinical features, and expression of ki-67, cyclin D1, p53 and p21^WAF1/Cip1^ was calculated by *χ*^2^ test. Survival curves were obtained by Kaplan-Meier analysis. In all cases, a *P* value <0.05 was considered to indicate statistical significance. Statistical analysis was performed using the SPSS software program 17.0 (SPSS Inc., Chicago, IL, USA).

## Results

### Expression of PRO2000/ANCCA and its correlation with clinicopathological characteristics in HCC

**Figure 1:**
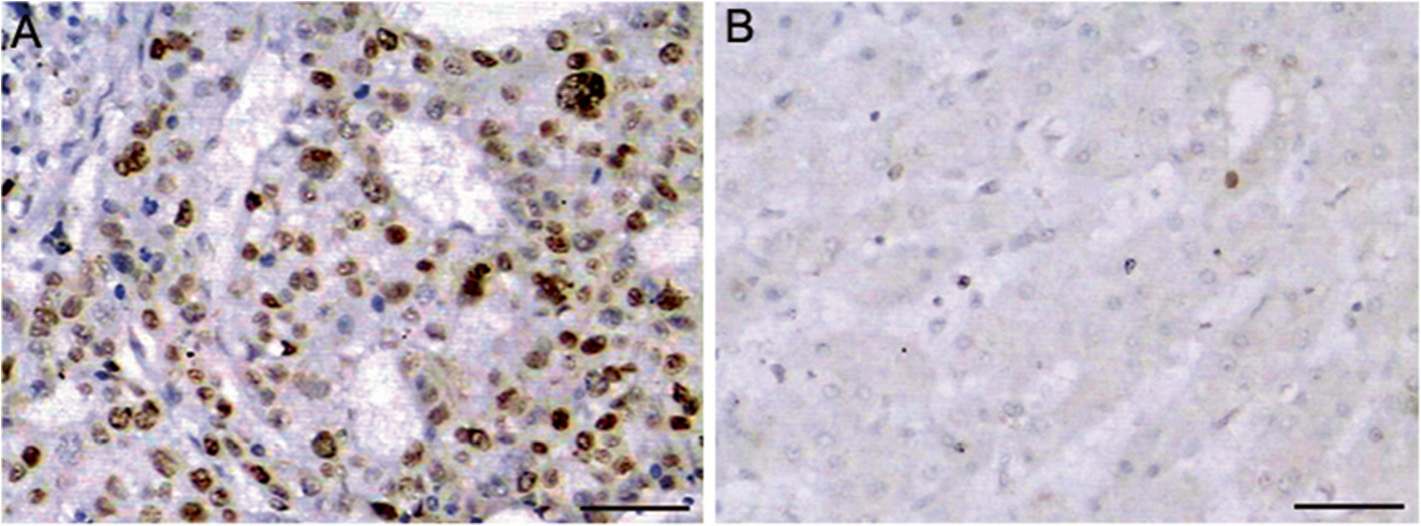
**Immunohistochemical staining of PRO2000/ANCCA protein in human liver tissue samples. (A)** HCC tissues and **(B)** adjacent non-tumor tissues were immunohistochemically stained with an anti-PRO2000/ANCCA antibody Positive PRO2000/ANCCA immunostaining was mainly localized in the nucleus of cells. Scale bars = 50 μm.

Immunohistochemical staining demonstrated a granular staining pattern of PRO2000/ANCCA in the nucleus of cancer cells (Figure 1A), whereas few scattered positive cells were detected in adjacent non-tumor tissues (Figure 1B). Sixty-six of 107 (61.68%) HCC specimens were presented positive staining for PRO2000/ANCCA, while 8 (7.48%) in non-cancer cells. It was positive in 30/31 (96.77%) poorly differentiated tumors and 36/76 (47.37%) well differentiated lesions. There was a significantly increased expression of PRO2000/ANCCA in poorly differentiated tumors than that in well differentiated tumors (*χ*^2^ = 36.736, *P* < 0.001). The intensity of PRO2000/ANCCA staining in cancer cells was much stronger than that in non-cancer cells.

**Figure 2:**
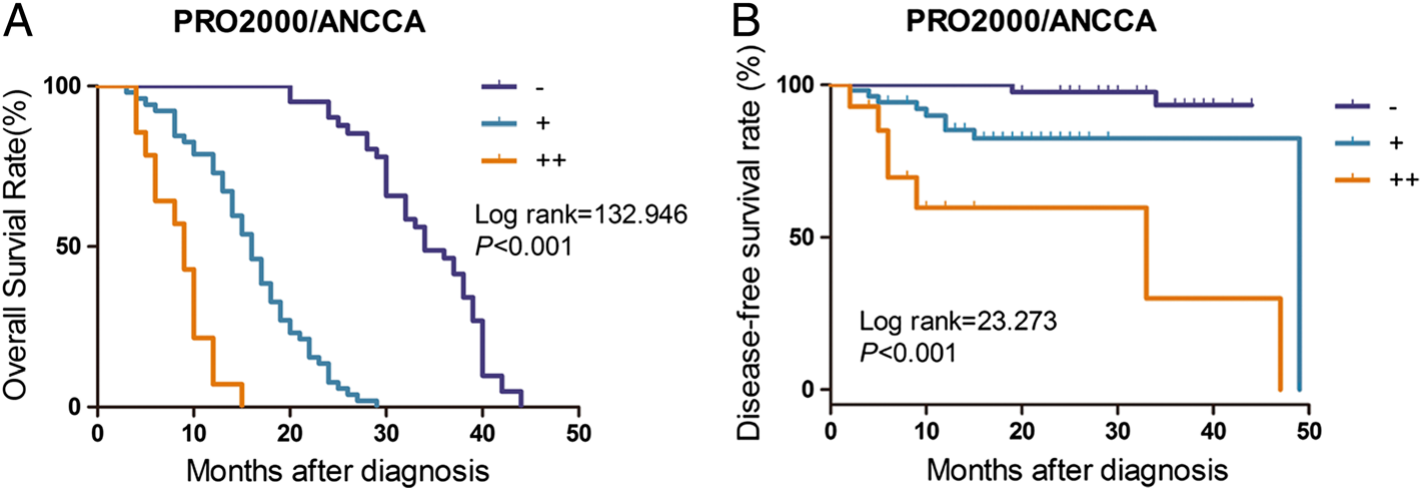
**Kaplan-Meier analyses of overall survival (A) and disease-free survival (B) in 107 HCC patients based on PRO2000/ANCCA expression.** –, negative; +, moderate positive; ++, strong positive.

We further analyzed the relationship between the expression of PRO2000/ANCCA and clinicopathological features of the HCC patients. As shown in Table 1, the expression of PRO2000/ANCCA was showed significant correlation with number of tumor nodules, TNM stage, tumor microsatellite, portal vein tumor thrombus and recurrence. However, no significant relationship was seen between PRO2000/ANCCA expression and gender, age, tumor size, cirrhosis, HBV infection and serum AFP level. In addition, Kaplan-Meier analysis and log-rank test revealed that high expression of PRO2000/ANCCA was significantly associated with shorter overall survival (Figure 2A) and disease-free survival (Figure 2B). Further multivariate Cox regression analysis indicated that PRO2000/ANCCA is an independent prognostic factor for survival of patients with HCC (*P* < 0.001, Table 2).

**Table 2:** Univariate and multivariate analyses of potential prognostic factors associated with overall survival of HCC patients

**Variables**	**Univariate analysis**		**Multivariate analysis**	
	**HR (95% CI)**	**P**	**HR (95% CI)**	**P**
**Gender** (male/female)	1.128 (0.602-2.115)	0.707		
**Age (years)** (≤51/>51)	2.305 (1.525-3.484)	<0.001	3.570 (2.311-5.516)	<0.001
**Serum HBsAg** (negative/positive)	0.822 (0.556-1.215)	0.326		
**Serum AFP (ng/ml)** (≤20/>20)	0.945 (0.645-1.384)	0.772		
**Tumor size (cm)** (≤5/>5)	1.410 (0.958-2.077)	0.082		
**Tumor number** (single/multiple)	2.250 (1.422-3.559)	0.001	1.149 (0.300-4.403)	0.839
**Tumor differentiation** (I-II/III-IV)	7.922 (4.739-13.242)	<0.001	3.292 (1.563-6.937)	0.002
**Cirrhosis** (absent/present)	0.999 (0.679-1.471)	0.996		
**TNM stage** (I-II/III-IV)	23.349 (11.008-49.527)	<0.001	3.848 (1.494-9.915)	0.005
**Tumor microsatellite** (absent/present)	1.877 (1.216-2.896)	0.004	1.404 (0.392-5.030)	0.602
**Portal vein tumor thrombus** (absent/present)	46.801 (16.166-135.495)	<0.001	11.741 (3.503-39.355)	<0.001
**Recurrence** (absent/present)	2.739 (1.628-4.609)	<0.001	1.220 (0.645-2.308)	0.541
**ANCCA expression** (−/+/++)	10.275 (6.440-16.395)	<0.001	8.745 (4.936-15.493)	<0.001

*HR*: hazard ratio, *CI* confidence interval. The P-value was calculated by Cox proportional hazards model.

### Association between PRO2000/ANCCA and ki-67 or cyclin D1 in HCC

**Figure 3:**
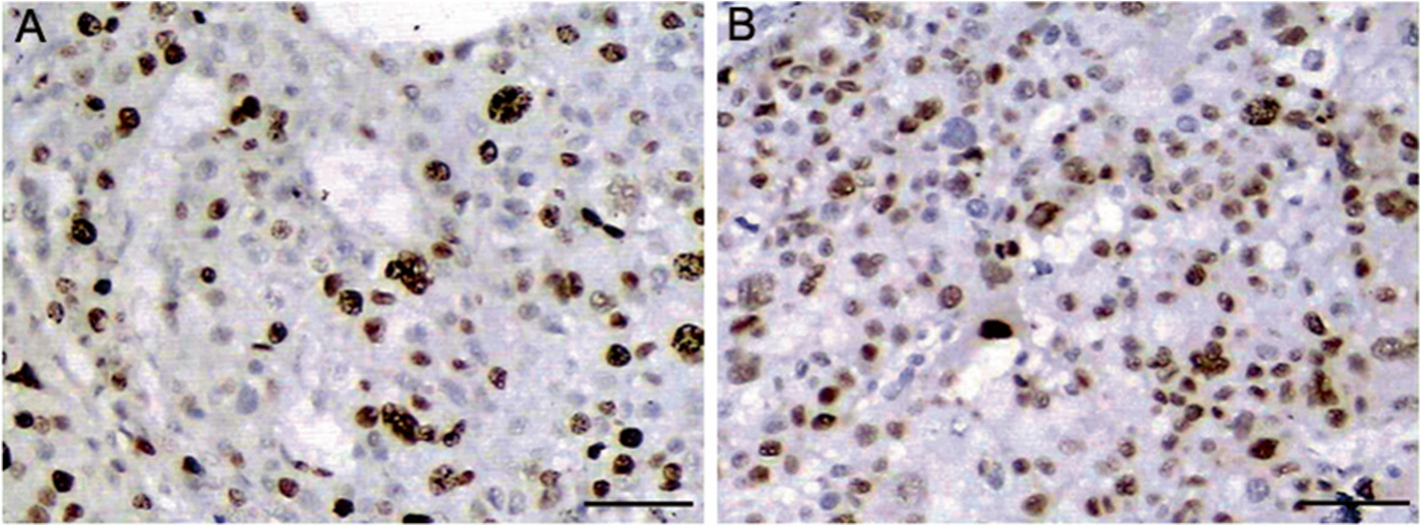
**Immunohistochemical expressions of ki-67 and cyclin D1 in consecutive sections of an HCC tissue.** Positive immunostaining of **(A)** ki-67 and **(B)** cyclinD1 were localized in the nucleus of cancer cell. Scale bars = 50 μm.

In all 107 cases of HCC, ki-67 and cyclin D1 positive cells were showed brown staining in the nucleus. In consecutive sections of an HCC, PRO2000/ANCCA, Ki-67 and cyclin D1 were showed to be co-expressed in the same cancer cell within individual tumor (Figure 3).

The correlation between PRO2000/ANCCA and ki-67 or cyclin D1 is summarized in Table 3. The ki-67 and cyclin D1 were significantly higher in PRO2000/ANCCA -positive group than that in PRO2000/ANCCA-negative group (*P* < 0.05).

**Table 3:** Relationship between PRO2000/ANCCA and ki-67, cyclin D1, p53 and p21 in HCC

**PRO2000/ANCCA**	**Cases**	**ki-67**		**cyclin D1**		**p53**		**p21**	
		**+**	**-**	**+**	**-**	**+**	**-**	**+**	**-**
Positive	65	46	19	40	25	29	36	26	39
Negative	42	18	24	9	33	25	17	13	29
*χ*^2^		8.270		16.536		2.269		0.902	
*P*-value		**0.004**		**<0.001**		0.132		0.342	

-, negative; +, positive. Significant differences are shown in bold.

### Association between PRO2000/ANCCA and p53 or p21 in HCC

**Figure 4:**
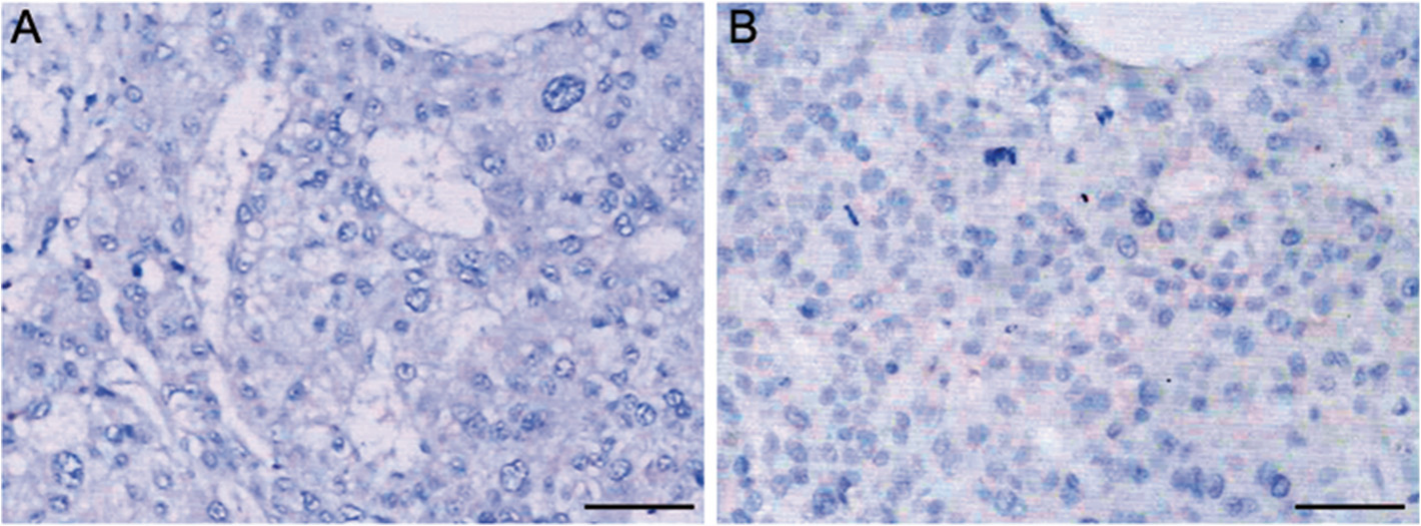
**Immunohistochemical staining of P53 and p21**^**WAF1/Cip1 **^**in consecutive sections of an HCC tissue.** Tissue sections were immunohistochemically stained with **(A)** anti-P53 and **(B)** anti-P21 antibodies. Scale bars = 50 μm.

Previous studies and our results showed nuclear staining for p53 or p21^WAF1/Cip1^ in HCCs. However, PRO2000/ANCCA expression was not consistent with p53 or p21^WAF1/Cip1^ in cancer cells in consecutive sections of an HCC (Figure 4). Nuclear immunoreactivity for p53 and p21 was found in 33 (42.86%) and 37 (48.05%) HCCs respectively. We analyzed the expression level of PRO2000/ANCCA and p53 and p21^WAF1/Cip1^ expression in HCC. Among PRO2000/ANCCA-positive cases, p53 and p21^WAF1/Cip1^ were expressed in 72.7% and 81.8% in HCC, respectively. In contrast to cases with negative PRO2000/ANCCA, p53 and p21^WAF1/Cip1^ were expressed in 72.4% and 79.3% in HCC. PRO2000/ANCCA expression was not significantly correlated with p53 and p21^WAF1/Cip1^ (Table 3).

## Discussion

PRO2000/ANCCA which located within a region of chromosome 8q was firstly reported to be an important candidate gene in HCC [3]. To our knowledge, investigation on biological function of PRO2000/ANCCA in HCC has not been reported. In this study, we firstly performed immunohistochemisty method to examine PRO2000/ANCCA expression in 107 cases of human HCC along with corresponding non-tumor tissues. The results showed that PRO2000/ANCCA protein was overexpressed in the majority of cancer cells comparing with surrounding hepatocytes. Our data are consistent with the preliminary report by Crawley et al. using comparative genomic microarray analysis. Of 107 HCC specimens, 61.68% were demonstrated positive staining for PRO2000/ANCCA, while 7.48% (8/107) in adjacent non-tumor tissues. There was a statistical difference of PRO2000/ANCCA expression in HCC and that in adjacent non-tumor tissues. The intensity of PRO2000/ANCCA expression in tumor cells was stronger than that in non-tumor cells. This result therefore supports the view that PRO2000/ANCCA may have specific function in malignant differentiation and proliferation of hepatocytes.

Our data also provided new insights regarding the relationship between PRO2000/ANCCA expression and clinicopathological features in HCC. There was a statistical difference of PRO2000/ANCCA expression in well differentiated tumors and that in poorly differentiated tumors (*P* < 0.05). Thus, level of PRO2000/ANCCA expression was significantly correlated with more aggressive tumor features such as number of tumor nodules, portal vein invasion, tumor microsatellite formation and TNM stage. Our study showed that PRO2000/ANCCA-positive cases had less survival rate than those in PRO2000/ANCCA-negative cases. These data suggest that PRO2000/ANCCA in HCC might serve as a valuable predictor for progression and poor prognosis.

PRO2000/ANCCA protein has been reported to be associated directly with estrogen-bound estrogen receptor α (ERα) and to mediate E2-stimulated expression of key cell cycle regulators likely via its ATP-driven protein complex remodeling function in breast cancer [7]. In this study, we observed the expression of PRO2000/ANCCA, Ki-67 and Cyclin D1 in series of sections and found these proteins were expressed in same cancer cell nucleus. Our results showed that PRO2000/ANCCA was strongly positive associated with ki-67 and cyclin D1 in HCC. As a proliferation-associated nuclear antigen, Ki-67 is present in cells that are replicating in the G1, S, G2, and M stages of the cell cycle [14]. Cyclin D1 functions as a regulatory subunit of CDK4 or CDK6, whose activity is required for cell cycle G1/S transition [15]. This finding might support the hypothesis that PRO2000/ANCCA may be involved in the regulation of proliferation in HCC.

The p53 tumor-suppressor gene has been shown to play a key role in the control of the cell cycle, cell differentiation and apoptosis. P21^WAF1/Cip1^, an inhibitor of cyclin-dependent kinases, is activated through p53-dependent or p53-independent pathway and plays an important role in regulation of the cell cycle, especially in G1 arrest [15-22]. Many studies have demonstrated that P53 and P21^WAF1/Cip1^ are involved in carcinogenesis of hepatocytes [23-25]. According to our results stated above, we are also interested in the relationship between PRO2000/ANCCA and p53 or P21^WAF1/Cip1^. In the present study, PRO2000/ANCCA expression was not consistent with p53 or P21^WAF1/Cip1^ in cancer cells within individual tumor. There was not any correlation between PRO2000/ANCCA and p53 or P21^WAF1/Cip1^. These results suggest that PRO2000/ANCCA might promote cell proliferation in HCC through a p53/ P21^WAF1/Cip1^-independent pathway.

In summary, this study demonstrated that PRO2000/ANCCA was overexpressed in HCC and was associated with clinicopathological features such as number of tumor nodules, TNM stage, tumor microsatellite, portal vein tumor thrombus and recurrence, but not with gender, age, cirrhosis, HBV infection and serum AFP level. There was a strong positive correlation between PRO2000/ANCCA expression and ki-67 and cyclin D1 but not p53 and p21. Our findings suggested that PRO2000/ANCCA may be involved in cell cycle regulation in the pathogenesis of HCC and serves as a predictor for poor prognosis of HCC. PRO2000/ANCCA might be a candidate gene for the development of diagnostic and therapeutic strategies for HCC. Further molecular, cellular and animal model studies are necessary to better understand the function of PRO2000/ANCCA as the present clinical samples study.

## Abbreviations

HCC: Hepatocellular carcinoma; AFP: Alpha fetal protein; ANCCA: AAA nuclear coregulator cancer-associated protein.

## Competing interests

The authors declare that they have no competing interests.

## Authors’ contributions

JY and JH performed clinical data acquisition, statistical analysis and drafted the manuscript. LL and ZC performed immunohistochemical study. YG performed critical revision. LG designed and directed the study. All authors read and approved the final manuscript.
